# Supplementary material for: The Systems Biology Research Tool: evolvable open-source software
Source: BMC Syst Biol. 2008 Jun 29;2:55. doi: 10.1186/1752-0509-2-55 (PMC2446383; doi:10.1186/1752-0509-2-55)
Supplement: Additional file 1 — SBRT Archive. An archive of the current version of the Systems Biology Research Tool. [file 1752-0509-2-55-S1.zip › sbrt-1.4.0/doc/developers_guide/api/sbrt/kernel/math/geom/comp/Taxicab.html]

Taxicab


|  |  |  |
| --- | --- | --- |
| **Overview** \| **Package** \| **Class** \| **Use** \| **Tree** \| **Deprecated** \| **Index** \| **Help** | |  |
| **PREV CLASS**   NEXT CLASS | **FRAMES**    **NO FRAMES**     **All Classes** |  |
| SUMMARY: NESTED \| FIELD \| CONSTR \| METHOD | DETAIL: FIELD \| CONSTR \| METHOD |  |


---


## sbrt.kernel.math.geom.comp Class Taxicab

```
java.lang.Object
  sbrt.kernel.math.geom.comp.Taxicab
```

---

``` public final class Taxicab extends java.lang.Object ```

This class is used to compute Taxicab distances. See Wikipedia for more
information. All provided numbers are converted to double precision via
the method `Number.doubleValue()`.

**Author:**
:   This class was written and documented by Jeremiah Wright while in the
    Wagner lab.

---

**Constructor Summary**

`Taxicab()`


| **Method Summary** | |
| --- | --- |
| `double` | `getDist(java.util.Collection<? extends java.lang.Number> x, java.util.Collection<? extends java.lang.Number> y)`             Computes and returns the Taxicab distance between the provided collections. |
| `double` | `getDist(java.util.Map<?,? extends java.lang.Number> x, java.util.Map<?,? extends java.lang.Number> y)`             Computes and returns the Taxicab distance between the provided maps. |
| `double` | `getDist(java.lang.Number[] x, java.lang.Number[] y)`             Computes and returns the Taxicab distance between the provided arrays. |

**Methods inherited from class java.lang.Object**

`clone, equals, finalize, getClass, hashCode, notify, notifyAll, toString, wait, wait, wait`

**Constructor Detail**

### Taxicab

```
public Taxicab()
```


**Method Detail**

### getDist

```
public double getDist(java.lang.Number[] x,
                      java.lang.Number[] y)
```

:   Computes and returns the Taxicab distance between the provided arrays.

    :   **Parameters:**: `x` - an array of numbers.: `y` - another array of numbers. **Returns:**: the Taxicab distance between the provided arrays. **Throws:**: `java.lang.IllegalArgumentException` - if the lengths of the provided arrays are not equal.

---


### getDist

```
public double getDist(java.util.Collection<? extends java.lang.Number> x,
                      java.util.Collection<? extends java.lang.Number> y)
```

:   Computes and returns the Taxicab distance between the provided collections.
    Numbers are compared upon iteration over each collection.

    :   **Parameters:**: `x` - a collection of numbers.: `y` - another collection of numbers. **Returns:**: the Taxicab distance between the provided collections. **Throws:**: `java.lang.IllegalArgumentException` - if the lengths of the provided collections are not equal.

---


### getDist

```
public double getDist(java.util.Map<?,? extends java.lang.Number> x,
                      java.util.Map<?,? extends java.lang.Number> y)
```

:   Computes and returns the Taxicab distance between the provided maps.
    The sets of keys from each map must be equal.

    :   **Parameters:**: `x` - a map.: `y` - another map. **Returns:**: the Taxicab distance between the provided maps. **Throws:**: `java.lang.IllegalArgumentException` - if the lengths of the provided maps or the key sets are not equal.


---


|  |  |  |
| --- | --- | --- |
| **Overview** \| **Package** \| **Class** \| **Use** \| **Tree** \| **Deprecated** \| **Index** \| **Help** | |  |
| **PREV CLASS**   NEXT CLASS | **FRAMES**    **NO FRAMES**     **All Classes** |  |
| SUMMARY: NESTED \| FIELD \| CONSTR \| METHOD | DETAIL: FIELD \| CONSTR \| METHOD |  |


---
